# Commentary: Post-Transplantation Cyclophosphamide Uniquely Restrains Alloreactive CD4+ T-Cell Proliferation and Differentiation After Murine MHC-Haploidentical Hematopoietic Cell Transplantation

**DOI:** 10.3389/fimmu.2022.887648

**Published:** 2022-04-14

**Authors:** Jessica Stokes, Richard J. Simpson, Emmanuel Katsanis

**Affiliations:** ^1^ Department of Pediatrics, University of Arizona, Tucson, AZ, United States; ^2^ Department of Immunobiology, University of Arizona, Tucson, AZ, United States; ^3^ The University of Arizona Cancer Center, Tucson, AZ, United States; ^4^ School of Nutritional Sciences and Wellness, University of Arizona, Tucson, AZ, United States; ^5^ Department of Medicine, University of Arizona, Tucson, AZ, United States; ^6^ Department of Pathology, University of Arizona, Tucson, AZ, United States

**Keywords:** haploidentical hematopoietic stem cell transplantation, graft versus host disease (GVHD), chemotherapy, murine (mouse), graft (regulation)

In their recent article Hadjis et al. investigated whether post-transplant cyclophosphamide (PT-CY) is unique in protecting against graft-versus-host disease (GvHD) by comparing it to multiple other “optimally dosed” chemotherapeutics (1). They concluded that PT-CY was superior to all other agents in ameliorating both clinical and histopathological GvHD. We posed analogous questions when we were the first to report on the use of post-transplant bendamustine (PT-BEN) as an alternative agent that could replace PT-CY (2). In contrast to the Hadjis et al. study, we demonstrated that PT-BEN was equally effective in preventing early GvHD and protecting against late GvHD, while enabling superior graft-versus-leukemia (GvL), when compared to PT-CY in murine haploidentical bone marrow transplantation (haplo-BMT) (2).

We would like to use this forum to discuss these discordant results. Firstly, the murine haploidentical models used in the two studies have noteworthy differences. In the Hadjis et al. report, a B6C3F1/Crl (H-2^b/k^) → B6D2F1/Crl (H-2^b/d^) mouse model was used. Recipient mice were conditioned with 10.5 Gy of single dose total body irradiation (TBI), were given 10^7^ bone marrow cells along with 4x10^7^ splenocytes (SC) on the same day (day 0), and received levofloxacin in their drinking water (1). We used a CB6F1 (H-2^d/b^) → CAF1/J (H-2^d/a^) model with recipient mice conditioned on day -1 with 6 or 10 Gy of TBI and infused 10^7^ donor bone marrow cells along with 3x10^7^ SC without prophylactic antibiotics (2). There are inherent strain combination variabilities in type, severity, and immunopathology of GvHD and the higher TBI dose, increased splenocyte dose, and use of prophylactic antibiotics altering gut microbiome would favor more severe GvHD in the Hadjis et al. haplo-BMT model compared to ours (3–6). Secondly, we gave PT-BEN only on day + 3 as we found that a single dose was more effective than a split dose and, importantly, it was administered intravenously (i.v.), using a different diluent. We injected PT-BEN i.v. because it is a known irritant/vesicant that has been reported to cause peritoneal sarcomas or fibrosarcomas when given intraperitoneally (i.p.) in certain strains of mice (7). In humans, vesicant agents given intraperitoneally are known to cause adhesions and trigger abdominal pain. Interestingly, Hadjis et al. gave PT-BEN i.p. and indicated that their optimal dose was 10 mg/kg given on days +3 and +4 compared to our 30 mg/kg i.v. single dose. Even with tail vein injections, these irritant/vesicant properties were evident, as we repeatedly observed that PT-BEN had substantial local irritation and in some cases loss of tails distally to the injection site when extravasation occurred during administration. Additionally, the doses of PT-CY differed in the two studies with 25 mg/kg versus 75 mg/kg given on days +3 and +4 i.p. Our higher dosing corresponded to 40% of the maximum tolerated dose (MTD) for both BEN and CY in our recipient mouse strain. To our knowledge, the MTD for BEN and CY in Hadjis et al’s recipient model is unknown, so it is unclear how the doses they used compare to ours. There is no data directly comparing an optimal i.v. dose of BEN to the PT-CY dosing optimized by Kanakry’s team in their model.

While in our studies we did observe histopathological evidence of liver GvHD in PT-BEN treated mice, equivalent to that seen with PT-CY, we did not observe the acsites with PT-BEN as reported by Hadjis et al. who stated that “the higher mean body weight in BEN-treated mice was a result of substantial weight gain from ascites, rather than clinical benefit from the chemotherapeutic and that autopsy suggested that these mice were developing ascites secondary to GVHD-induced liver failure and protein-losing enteropathy”. It is not clear how liver failure was confirmed as particular to PT-BEN as this drug does not have specific liver toxicity and severe GvHD was present with many of the other agents tested. It is also intriguing that the mice that received the chemotherapeutic agents with vesicant or irritant properties (BEN, VCR, PTX) all had higher liver GvHD histopathologic scores when compared to those receiving non-vesicants (CY, MTX, ARA-C), which raises the question of whether direct local toxicity to peritoneum and intraperitoneal organs, such as the liver, may have been a contributing factor, especially in the post-TBI setting. Moreover, the small and large intestine histopathologic scores were similar in recipients receiving BEN, VCR, PTX and ARA-C. It is thus unexpected that only PT-BEN recipients developed protein-losing enteropathy. It is also possible that cytokine release syndrome (CRS)/capillary leak, rather than GvHD alone, may have contributed to the ascites observed with PT-BEN.

A strategy being explored to reduce relapse rates, due to suppressed GvL, is a dose reduction of PT-CY. Preclinical studies in mismatched murine BMT models by Kanakry et al. have demonstrated benefits of reducing the PT-CY dose (8). This led to a clinical trial by the same investigator at the NIH (NCT03983850) evaluating de-escalation of PT-CY to half the dose (25 mg/kg on days +3 and +4) of current standard. Presentation of their early findings indicated that a reduction in the dose of PT-CY maintained protection against severe acute GVHD, while promoting more rapid engraftment and decreasing early post-transplant toxicity (9).

A body of literature has only recently started to emerge, in large part from our group (2, 10–14), regarding the immunomodulatory properties of BEN on MDSCs, DC subsets, and T cells (10–14). In all our murine models, whether BEN is given pre- or post-transplant, it has been associated with decreased GvHD and increased GvL. Our extensive preclinical work has provided the foundation to explore a different approach than simply reducing the dose of PT-CY. Our first in human Phase Ia clinical trial (NCT02996773) was a 3 + 3 dose escalation trial, which accrued pediatric and young adult patients with hematologic malignancies. In our interim analysis, we reported that PT-CY/BEN was well tolerated with early engraftment and no dose limiting toxicities (15). Hadjis et al. stated that post-transplantation BEN has shown mixed results in early phase clinical trials citing our Phase Ia (15) and a recent report by Moiseev et al. (16). These studies both documented early engraftment and reduction in aGvHD. However, the Moiseev et al. study was complicated by cytokine release syndrome (CRS) after PT-BEN in 70% of their patients (16). Of significance, however, is that their study population consisted of older patients with advanced refractory hematologic malignancies who received much higher doses of PT-BEN (total dose 140-280 mg/m^2^) and no concurrent PT-CY. Additionally, 44% of their patients did not receive tacrolimus (Tacro) or mycophenolate mofetil (MMF) and 85% received peripheral blood stem cell (PBSC) rather than bone marrow grafts, which is known to elicit more severe GvHD. CRS was not observed in our first three cohorts who received lower doses of PT-BEN (20-90 mg/m^2^) in combination with PT-CY, Tacro and MMF but did occur in one of three patients (Cohort 4) who received a dose of PT-BEN totaling 110 mg/m^2^ (20 mg/m^2^ day +3 and 90 mg/m^2^ day +4) given following day +3 PT-CY (40 mg/kg). Taken together, this might indicate a) a dose dependent effect on risk of CRS with escalation of PT-BEN, b) a protective effect against CRS when PT-BEN is used following PT-CY and in conjunction with Tacro and MMF, c) CRS may be more common with PBSC grafts and/or d) CRS may be more frequent in patients transplanted with relapsed refractory disease.

We appreciate the many contributions of Kanakry and colleagues to our understanding of the mechanisms by which PT-CY reduces GvHD in both pre-clinical and clinical studies. However, it is imperative to always seek to thoughtfully explore potential improvements in current therapeutic approaches and the PT-CY arena is no exemption. It is important to highlight that the trial by Moiseev et al. (16) was sparked by our preclinical studies (2) and mirrored them as they completely replaced PT-CY with PT-BEN and initially did not combine it with Tacro and/or MMF. They soon discovered the necessity to de-escalate their dose and add additional GvHD prophylaxis. In stark contrast, we did not directly translate our own preclinical approach, but instead very cautiously designed our Phase Ia to explore progressive replacement of PT-CY starting with day +4 and given in combination with Tacro and MMF (15). Our trial continues as a Phase Ib without further escalation of PT-BEN to confirm the safety and assess potential benefits of day +3 PT-CY 50 mg/kg with day +4 PT-BEN 90 mg/m^2^ compared to PT-CY alone with a thorough investigation of immune reconstitution. We contend that combining PT-CY/BEN remains a promising treatment option that necessitates continued rigorous and cautious evaluation.

## Author Contributions

All authors listed have made a substantial, direct, and intellectual contribution to the work and approved it for publication.

## Conflict of Interest

The authors declare that the research was conducted in the absence of any commercial or financial relationships that could be construed as a potential conflict of interest.

## Publisher’s Note

All claims expressed in this article are solely those of the authors and do not necessarily represent those of their affiliated organizations, or those of the publisher, the editors and the reviewers. Any product that may be evaluated in this article, or claim that may be made by its manufacturer, is not guaranteed or endorsed by the publisher.

## References

[1] Hadjis AD Nunes NS Khan SM Fletcher RE Pohl A Venzon DJ. Post-Transplantation Cyclophosphamide Uniquely Restrains Alloreactive CD4+ T-Cell Proliferation and Differentiation After Murine MHC-Haploidentical Hematopoietic Cell Transplantation. Front Immunol (2022) 13:796349. doi: 10.3389/fimmu.2022.796349 35242129 PMC8886236

[2] Stokes J Hoffman EA Zeng Y Larmonier N Katsanis E. Post-Transplant Bendamustine Reduces GvHD While Preserving GvL in Experimental Haploidentical Bone Marrow Transplantation. Br J Haematol (2016) 174(1):102–16. doi: 10.1111/bjh.14034 PMC4917459 27030315

[3] Ziegler M Han JH Landsburg D Pegues D Reesey E Gilmar C. Impact of Levofloxacin for the Prophylaxis of Bloodstream Infection on the Gut Microbiome in Patients With Hematologic Malignancy. Open Forum Infect Dis (2019) 6(7):ofz252. doi: 10.1093/ofid/ofz252 31281857 PMC6602896

[4] Routy B Letendre C Enot D Chenard-Poirier M Mehraj V Seguin NC. The Influence of Gut-Decontamination Prophylactic Antibiotics on Acute Graft-Versus-Host Disease and Survival Following Allogeneic Hematopoietic Stem Cell Transplantation. Oncoimmunology (2017) 6(1):e1258506. doi: 10.1080/2162402X.2016.1258506 28197380 PMC5283637

[5] Socie G Kean LS Zeiser R Blazar BR. Insights From Integrating Clinical and Preclinical Studies Advance Understanding of Graft-Versus-Host Disease. J Clin Invest (2021) 131(12):4327–36. doi: 10.1172/JCI149296 PMC8203454 34101618

[6] Arber C Brenner MK Reddy P. Mouse Models in Bone Marrow Transplantation and Adoptive Cellular Therapy. Semin Hematol (2013) 50(2):131–44. doi: 10.1053/j.seminhematol.2013.03.026 PMC3971726 24216170

[7] Product Monograph -Treanda (Bendamustine Hydrochloride for Injection) (2018). Available at: https://pdf.hres.ca/dpd_pm/00043152.PDF.

[8] Wachsmuth LP Patterson MT Eckhaus MA Venzon DJ Kanakry CG. Optimized Timing of Post-Transplantation Cyclophosphamide in MHC-Haploidentical Murine Hematopoietic Cell Transplantation. Biol Blood Marrow Transplant (2020) 26(2):230–41. doi: 10.1016/j.bbmt.2019.09.030 PMC7590501 31586477

[9] McAdams MJ Hyder M Dimitrova D Sadler JL Christi McKeown C Steinberg SM. (2021). Phase I/II Study of Reduced Dosing of Post-Transplantation Cyclophosphamide (PTCy) After HLA-Haploidentical Bone Marrow Transplantation. In: Presented American Society of Hematology Annual Meeting. Available at: https://ash.confex.com/ash/2021/webprogram/Paper146997.html.

[10] Molina MS Stokes J Hoffman EA Eremija J Zeng Y Simpson RJ. Bendamustine Conditioning Skews Murine Host DCs Toward Pre-Cdc1s and Reduces GvHD Independently of Batf3. Front Immunol (2020) 11:1410. doi: 10.3389/fimmu.2020.01410 32765499 PMC7378358

[11] Stokes J Hoffman EA Molina MS Kummet N Simpson RJ Zeng Y. Bendamustine With Total Body Irradiation Conditioning Yields Tolerant T-Cells While Preserving T-Cell-Dependent Graft-Versus-Leukemia. Oncoimmunology (2020) 9(1):1758011. doi: 10.1080/2162402X.2020.1758011 32391190 PMC7199810

[12] Stokes J Hoffman EA Molina MS Eremija J Larmonier N Zeng Y. Bendamustine With Total Body Irradiation Limits Murine Graft-Versus-Host Disease in Part Through Effects on Myeloid-Derived Suppressor Cells. Biol Blood Marrow Transplant (2019) 25(3):405–16. doi: 10.1016/j.bbmt.2018.10.009 PMC7571287 30326280

[13] Stokes J Molina MS Hoffman EA Simpson RJ Katsanis E. Immunomodulatory Effects of Bendamustine in Hematopoietic Cell Transplantation. Cancers (Basel) (2021) 13(7):1702. doi: 10.3390/cancers13071702 33916711 PMC8038415

[14] Molina MS Hoffman EA Stokes J Kummet N Smith KA Baker F. Regulatory Dendritic Cells Induced by Bendamustine Are Associated With Enhanced Flt3 Expression and Alloreactive T-Cell Death. Front Immunol (2021) 12:699128. doi: 10.3389/fimmu.2021.699128 34249005 PMC8264365

[15] Katsanis E Maher K Roe D Simpson RJ. Progressive Substitution of Post-Transplant Cyclophosphamide With Bendamustine: A Phase I Study in Haploidentical Bone Marrow Transplantation. eJHaem (2020) 1(1):286–92. doi: 10.1002/jha2.20 PMC9176108 35847727

[16] Moiseev I Bondarenko S Morozova E Vlasova Y Dotsenko A Epifanovskaya O. Graft-Versus-Host Disease Prophylaxis With Post-Transplantation Bendamustine in Patients With Refractory Acute Leukemia: A Dose-Ranging Study. Transplant Cell Ther (2021) 27(7):601.e1–7. doi: 10.1016/j.jtct.2021.03.032 33845259

